# Case report of an occipital neuralgia caused by a vertebral artery loop: surgical strategies from a rare neurovascular conflict

**DOI:** 10.3389/fsurg.2026.1817648

**Published:** 2026-05-28

**Authors:** Julien Haemmerli, Michele Pio Fabrizio, Arash Najafi, Andreas Grüner, Alex Alfieri

**Affiliations:** 1 Department of Neurosurgery and Spine Surgery, Cantonal Hospital of Winterthur, Winterthur, Switzerland; 2 Faculty of Medicine, University of Geneva, Geneva, Switzerland; 3 Department of Radiology and Nuclear Medicine, Cantonal Hospital Winterthur, Winterthur, Switzerland; 4 Institute for Anesthesiology and Pain Medicine, Hospital Buelach, Buelach, Switzerland; 5 Biomedical Faculty, University of Southern Switzerland (USI), Lugano, Switzerland

**Keywords:** C3 nerve root, case report, neurovascular compression, occipital neuralgia, posterior decompression, vertebral artery dominance

## Abstract

**Background:**

Occipital neuralgia secondary to cervical nerve root compression is most commonly associated with degenerative or post-traumatic foraminal stenosis. In contrast, neurovascular conflict caused by an aberrant vertebral artery loop represents a rare and underrecognized etiology. Optimal surgical management remains variable, with multiple surgical techniques with risks and level of invasiveness.

**Case description:**

A 74-year-old woman presented with a five-year history of severe, progressively worsening left-sided occipital neuralgia refractory to conservative treatment. The pain was characterized as sharp and lancinating, with paroxysmal, electric shock–like exacerbations, resulting in substantial impairment of quality of life. Imaging with MRI and CTA demonstrated a dominant left vertebral artery forming a foraminal loop at C2–C3, directly compressing the exiting C3 nerve root. Microsurgical decompression was performed through a posterior paramedian approach in a hybrid operating room setting. Although arterial transposition was initially considered, intraoperative findings revealed limited vessel mobility in the context of vertebral artery dominance, necessitating modification of the surgical strategy. Adequate osseous and neurovascular decompression was achieved, and an interpositional spacer was placed between the artery and the nerve root. Intraoperative angiography confirmed preserved arterial patency without evidence of flow compromise. At three months, the patient reported complete resolution of pain, with no new neurological deficits or signs of cervical instability.

**Conclusion:**

Aberrant vertebral artery loops should be considered in the differential diagnosis of refractory occipital neuralgia, particularly when conventional etiologies fail to explain persistent symptoms. Surgical treatment must be individualized and guided by intraoperative anatomical and functional findings. In selected cases, minimally invasive, targeted decompression without arterial transposition may achieve effective and durable symptom relief while reducing the risk of vascular injury and structural instability.

## 1 Introduction

Cervical radicular neuralgia is most commonly caused by disc herniation, degenerative changes with osteophyte formation, trauma, and more rarely by tumors (1). When the compression on the nerve, its root or both affects the nerve C1, C2 or C3, patients present pain either on the upper neck, the occipital region or both (2). These symptoms fall within the spectrum of occipital neuralgia, a broader group of conditions related to irritation or compression of the occipital nerves along their course from the root to their distal branches.

The vertebral arteries (VA) play a critical role in cerebral and spinal cord perfusion, supplying nearly 30% of the brain's blood flow and contributing to the vascularization of the anterior spinal cord. Anatomically, both VA usually ascend from the C6 level through the transverse foramina (V2 segment), then form a medial loop over the superior surface of the atlas (V3 segment) before entering the intradural space through the foramen magnum (V4 segment, or intradural) (3, 4). From V2 to V3, their course is generally straight but may be stretched with head and neck rotation.

Between two transverse foramina, the VA runs posterior and lateral to the exiting cervical nerve root. At this segment, aberrant arterial loop may compress the nerve root at its foraminal or extraforaminal portion, resulting in radicular pain (5–8). This rare form of neurovascular conflict is frequently overlooked, often resulting in delayed diagnosis, prolonged ineffective treatment, cross-border consultations, and the development of chronic neuropathic pain with significant psychosocial consequences.

This manuscript focuses on the direct surgical management of a C2–C3 neurovascular conflict, discussing a minimally invasive decompression technique that preserves the VA *in situ*, avoids arterial transposition, and does not require complete facetectomy or posterior fixation and fusion. This case report was performed according to the SCARE-Guidelines (9).

## 2 Case presentation

### 2.1 Presentation

A 74-year-old patient was referred to our neurosurgical outpatient clinic for severe, progressive left-sided occipital headache of approximately five years duration, radiating anteriorly from the occipital region toward the frontal area. The pain was described as sharp and lancinating, reaching a maximum intensity of 10/10 on the visual analog scale, predominantly at night, leading to significant sleep deprivation and a marked impairment in quality of life. The patient reported superimposed electric shock–like paroxysms. During particularly severe episodes, she had expressed suicidal ideation and had even registered with a Swiss assisted-suicide organization. Temporary symptom relief was achieved with daily off-label use of zolmitriptan nasal spray. Multiple conservative treatments provided either no or only short-term improvement.

Her medical history was notable for moderate mitral valve insufficiency, pharmacologically controlled arterial hypertension, hypothyroidism, BRCA2-mutated breast cancer, and prior lumbar spine decompression surgery.

Clinically, the pain distribution corresponded to the left C3 dermatomes, radiating from the upper cervical area to the occipital and frontal regions. Neurological examination revealed no motor weakness or sensory deficits. Cervical magnetic resonance imaging (MRI) with dedicated arterial sequences (MRA), combined with cranio-cervical computed tomography angiography (CT), demonstrated a left-sided VA loop at the C2–C3 foramen in direct contact with and compressing the exiting C3 nerve root (Figures 1, 2). The contralateral (right side) VA was hypoplastic, resulting in left-sided vascular dominance (ratio >2). Degenerative changes of the upper cervical facet joints were also noted but were considered insufficient to account for the severity of the symptoms.

**Figure 1:**
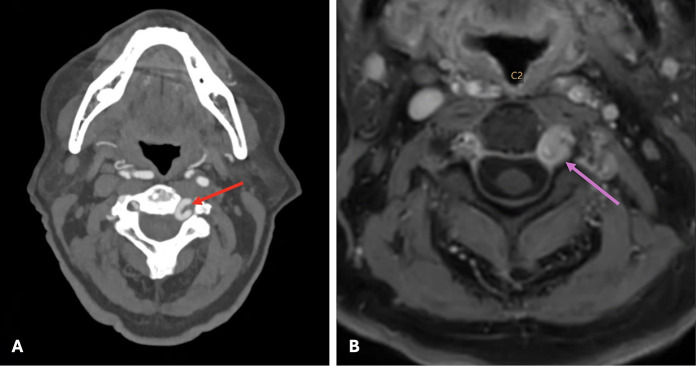
Preoperative vascular imaging demonstrating the vertebral artery loop. **(A)** Computed tomography angiography showing the aberrant vertebral artery loop within the left C2–C3 foramen (red arrow). **(B)** Magnetic resonance angiography demonstrating foraminal occupation by the arterial loop; the violet arrow indicates the exiting C3 nerve root.

**Figure 2:**
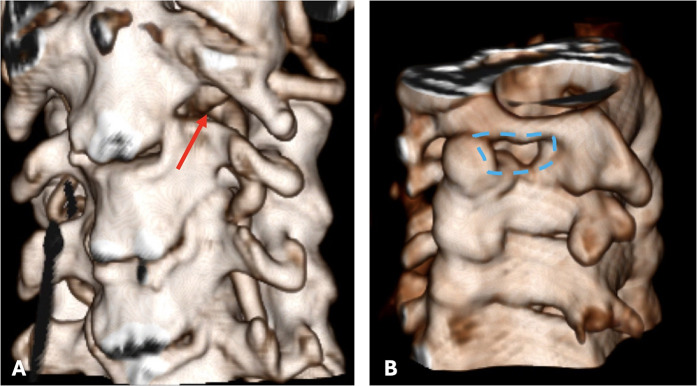
Three-dimensional computed tomography reconstruction of the upper cervical spine. **(A)** Preoperative left anterolateral view illustrating the position of the vertebral artery loop within the foramen (red arrow). **(B)** Postoperative posterior view showing the approximate size of the bony decompression window.

Given the clinical presentation consistent with C3 radicular neuralgia and the radiological evidence of a neurovascular conflict caused by an aberrant VA loop, microsurgical decompression at the left C2–C3 level was indicated. Surgery was planned via a paramedian posterior approach in a hybrid neurovascular operating room. In collaboration with the neurointerventional radiology team, VA loop mobilization and transposition with nerve root decompression was scheduled, with the support of an intraoperative angiogram.

### 2.2 Surgical procedure description

The patient was positioned prone in the hybrid operating room. Head fixation was achieved using a carbon fiber head clamp (DORO®, Black Forest Medical Group, Freiburg, Germany). Intraoperative fluoroscopy with a C-arm system (Pheno Artis®, Siemens, Erlangen, Germany) was used to accurately localize the C2–C3 level.

The left brachial artery was surgically exposed, and a 5F arterial sheath was inserted. Digital subtraction Angiography (DSA) was subsequently performed using a pigtail catheter positioned in the proximal left subclavian artery to visualize the VA before and after decompression. For the neurosurgical approach, a midline skin incision was made between the spinous processes of C2 and C3. After subperiosteal muscle dissection, the left C2/C3 laminae were exposed. Under the surgical microscope, a left-sided partial hemilaminectomy of C2 was performed and extended laterally toward the C3 neuroforamen, with careful preservation of the C2-C3 facet joint. The procedure was performed in a minimally invasive manner.

After targeted opening and decompressing the left C3 foramen (Figure 2), the VA loop was directly visualized within the foramen. The exiting nerve root appeared flattened and compressed against the inferior, lateral and dorsal margins of the foramen. Adequate bone decompression of C2 lamina was achieved to allow gentle mobilization of the nerve root. Microsurgical dissection was carried out along the inferior aspect of the arterial loop, and the nerve root was carefully separated from the vessel. Dense fibrosus tissue within the foraminal region required meticulous dissection to free the artery from both neural and osseous attachments.

Given the dominance of the left VA and the limited mobility of the loop, an intraoperative decision was made to preserve the artery *in situ* rather than proceed with transposition. Following adequate decompression of the neurovascular conflict, a small cigarette-shaped piece of absorbable hemostatic sponge (Spongostan®, Johnson & Johnson, New Brunswick, USA) (rolled 2 mm × 3 mm) was interposed between the artery and the nerve root to maintain separation (Figure 3). No vascular injury or intraoperative complications occurred. Final DSA confirmed preserved VA patency without evidence of stenosis or flow compromise.

**Figure 3:**
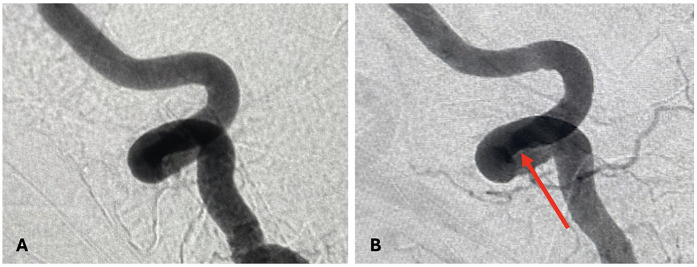
Digital subtraction angiography (DSA) of the vertebral artery loop. **(A)** Intraoperative pre-decompression DSA demonstrating the vertebral artery loop. **(B)** Intraoperative post-decompression DSA showing the vertebral artery loop after placement of the interpositional spacer. The red arrow indicates the site of spacer interposition between the artery and the nerve root, reflected by a subtle focal deformation of the arterial loop.

### 2.3 Postoperative course

No postoperative neurological deficits were observed. Dynamic cervical radiographs obtained six weeks after surgery demonstrated no evidence of segmental instability at the C2–C3 level.

At three-month follow-up, the patient reported complete resolution of her left-sided occipital pain and expressed high satisfaction with the surgical outcome, noting a substantial improvement in her quality of life.

## 3 Discussion

Occipital neuralgia is characterized by paroxysmal, lancinating pain in the distribution of the greater, lesser, or third occipital nerves, often accompanied by persistent background discomfort and local tenderness over the nerve's course (2, 10). The pain typically radiates from the upper cervical region toward the occiput and, in some cases, to the frontal or orbital areas via trigeminocervical convergence. Etiologies include entrapment, trauma, degenerative cervical changes, inflammatory processes, and, more rarely, neurovascular conflicts at the upper cervical root level (2). Beyond secondary structural causes, occipital neuralgia may also display familial clustering, suggesting a potential genetic or constitutional predisposition to cranial nerve hyperexcitability possibly involving aberrant neurovascular relationships or intrinsic susceptibility to ephaptic transmission (10).

Through this case report and surgical technical note, we describe a patient presenting with refractory cervicogenic occipital neuralgia caused by a rare neurovascular conflict: an aberrant VA loop within the left C3 foramen producing direct compression of the exiting C3 nerve root. Preoperatively, arterial mobilization and transposition were considered to achieve definitive decompression. However, intraoperative anatomy with limited vessel mobility and ipsilateral VA dominance prompted a strategy shift toward a targeted osseous and neurovascular decompression while preserving the artery *in situ*. Separation of the neurovascular interface was maintained using an interpositional spacer. This individualized, anatomy-driven approach resulted in complete pain relief, preserved cervical alignment, and no radiographic evidence of C2–C3 instability, while minimizing manipulation of a dominant VA.

### 3.1 Vertebral artery loop affecting cervical nerve and causing a neuralgia

Compression of a cervical nerve root by an aberrant VA loop has already been described in the literature (5, 6, 11–17). However, most available reports consist of isolated case reports or small technical notes involving one or two patients, highlighting the rarity of this condition compared with the far more common degenerative or traumatic causes of foraminal stenosis (5, 6, 8, 11–13, 16–19). In the present case, the diagnosis was initially suspected by the pain specialist after careful evaluation of cervical CT with angiographic sequences. Such vascular anomalies may easily be overlooked on routine imaging, particularly when attention is primarily directed toward degenerative changes.

Regarding its prevalence, Ekşi et al. retrospectively analyzed 230 cervical magnetic resonance (MR) angiograms (20) and identified VA loop in 13 patients (5,9%) affecting at least one cervical foramen. More recently, Semonche et al. reported a prevelance up to 5.9% (13), confirming Ekşi's results.The anomaly was more frequent in women, and in patients in the fifth to sixth decades of life. Not all patients presented with radiculopathy. Clinical manifestations ranged from neck pain to vertebrobasilar insufficiency, while some individuals remained asymptomatic. C4–C5 and secondary C3–4 are the most common affected levels (14).

These findings support the use of dedicated vascular imaging when a neurovascular conflict is suspected. Both CT angiography and MR angiography are useful modalities for identifying arterial loops and their relationship to the nerve root (20, 21). In our case, an intraoperative DSA was performed to analyze the exact shape of the loop, the flow inside the loop after decompression, and to confirm the patency of the affected VA.

### 3.2 Relationship to other neurovascular conflict syndromes

The clinical phenotype in our patient, lancinating baseline pain with superimposed electric shock–like paroxysms, shares descriptive overlap with classical neurovascular compression syndromes, most notably trigeminal neuralgia (22–24). However, the presumed pathophysiology differs in relevant ways. Trigeminal neuralgia is typically attributed to pulsatile neurovascular contact at the root entry zone, with ephaptic transmission and hyperexcitability generating brief, triggerable paroxysms (24). By contrast, in foraminal VA loop conflict the hypothesis relies on the fact that the nerve root may be subjected to a more constrained, mechanically disadvantageous environment: the artery occupies a fixed osseous corridor, producing direct root deformation and tethering. In such a setting, symptoms likely arise from a mixed mechanism: persistent mechanical compression with superimposed pulsatile irritation, potentially explaining the combination of sustained pain and intermittent “electrizing” attacks observed here. This distinction is clinically relevant because it influences both imaging strategy and surgical goals (restoration of space and mobility at the root within the foramen).

### 3.3 Surgical strategy: decompression, transposition, and the value of intraoperative adaptability

The operative objective in VA loop-related radicular occipital neuralgia is elimination of the neurovascular conflict while preserving vascular integrity and cervical stability. The posterior approach was chosen to allow direct access to the C2–C3 lamina and foramen with the intent to preserve the facet joint. The initial surgical plan consisted of opening the left C2–C3 foramen while preserving the facet joint, followed by microsurgical dissection of the VA loop and its transposition away from the nerve root. However, two findings argued against aggressive arterial repositioning: the dense fibrous adhesions limiting safe mobility and the functional dominance of the ipsilateral VA with a hypoplastic contralateral artery, amplifying the potential consequences of dissection, intimal injury, thrombosis, or embolic events. This scenario illustrates a key principle: in rare vascular variants, the preoperative plan must remain provisional, with the definitive strategy determined by real-time assessment of vessel mobility, dominance, and the safety margin for manipulation.

We therefore adopted a more conservative, targeted solution: adequate bony decompression of the foramen and microsurgical release of the root from the arterial loop, followed by placement of an interpositional spacer to maintain separation. This technique aims to achieve the functional equivalent of microvascular decompression by creating a durable buffer, without the risks inherent to transposition of a dominant VA. Several surgical strategies have been described for the treatment of this condition, including both direct and indirect decompression techniques. Direct decompression through posterior approach were described. Direct posterior decompression has been reported by multiple authors. Rho et al. described complete facetectomy with posterior pedicle screw fixation and reported favorable clinical outcomes in a single patient (21). Detwiler et al. proposed a similar decompression without the need for posterior fixation (15). Other groups described a similar technique as ours with the use of Teflon® as interpositional material (12). In the present case, an absorbable hemostatic Spongostan® was preferred in order to avoid permanent foreign material within the limited foraminal space. Anterolateral approaches have also been described, allowing direct foraminal decompression and providing greater freedom for VA mobilization or transposition (18, 19).

In contrast, indirect decompression through an anterior approach has been proposed by Farshad et al., who performed anterior release, distraction, and fusion with placement of an 8 mm interbody cage to straighten the VA (11).

### 3.4 Interposition material: permanence vs. adaptability

The choice of interpositional material is not trivial in the confined cervical foramen. Permanent implants may provide durable separation but also introduce long-term foreign body considerations in a tight neurovascular corridor. In this case, an absorbable hemostatic sponge was selected to minimize permanent material burden. While absorbable spacers may theoretically reduce long-term foreign body risk, they raise the question of whether separation persists after resorption. We would argue that, when combined with sufficient bony decompression and meticulous neurolysis, the spacer functions primarily as a short- to mid-term “separator” that facilitates stabilization of the new interface during early postoperative healing.

### 3.5 Stability-preserving decompression and risk mitigation

A central technical goal in our approach was to avoid destabilizing the C2–C3 motion segment. Complete facetectomy can enlarge the working corridor but increases the risk of postoperative instability, potentially necessitating fusion. Here, careful preservation of the facet joint combined with limited hemilaminectomy and foraminal enlargement provided adequate exposure while maintaining segmental integrity, as supported by dynamic radiographs at six weeks. Additionally, use of a hybrid operating room and intraoperative angiography provided a safety net for verifying patency after manipulation in a dominant vessel.

### 3.6 Clinical implications

This case underscores three practical points. First, VA loops should be actively considered in the differential diagnosis of refractory occipital neuralgia with radicular features, especially when routine imaging fails to explain symptom severity. Second, dedicated vascular imaging (CTA/MRA), supplemented by DSA when feasible, can clarify anatomy and enhance procedural safety. Third, surgical treatment should be individualized: in the setting of limited loop mobility and VA dominance, minimally access decompression with interposition without transposition, complete facetectomy, or fusion, can provide effective symptom relief while reducing vascular and structural risk.

### 3.7 Limitations

This report describes a single case, which limits generalizability and does not allow assessment of complication rates or comparison with alternative surgical strategies such as arterial transposition. In addition, the absence of a control group precludes conclusions regarding the relative efficacy of different decompression techniques. The long-term behavior and resorption kinetics of the absorbable interpositional material in this anatomical setting also remain uncertain and warrant further observation. Larger series with standardized reporting of surgical strategies, materials used, and long-term outcomes are needed to better define optimal management.

## 4 Conclusion

Aberrant vertebral artery loops represent a rare but clinically relevant cause of refractory cervical radicular occipital neuralgia. This entity should be considered when symptoms are severe, from dermatomes consistent, and disproportionate to degenerative findings on routine imaging. Dedicated vascular imaging is essential to establish the diagnosis and to define the anatomical relationship between the artery and the affected nerve root.

Surgical management must be individualized and guided by intraoperative findings, particularly vessel dominance, mobility, and the extent of foraminal constraint. In selected cases, targeted posterior decompression with preservation of the vertebral artery *in situ* and interpositional separation can achieve complete symptom relief while minimizing vascular manipulation, avoiding facetectomy and fusion, and preserving segmental stability.

## Data Availability

The raw data supporting the conclusions of this article will be made available by the authors, without undue reservation.
